# Evolution of glial cells: a non-bilaterian perspective

**DOI:** 10.1186/s13064-024-00184-4

**Published:** 2024-06-21

**Authors:** Larisa Sheloukhova, Hiroshi Watanabe

**Affiliations:** Evolutionary Neurobiology Unit, Okinawa Institute of Science and Technology, 1919-1 Tancha, Onna-son, Kunigami-gun, Okinawa, 904-0412 Japan

**Keywords:** Glia, Evolution, Glial cells missing, Non-bilaterians, Gliogenesis, Neurogenesis, Cnidaria

## Abstract

Nervous systems of bilaterian animals generally consist of two cell types: neurons and glial cells. Despite accumulating data about the many important functions glial cells serve in bilaterian nervous systems, the evolutionary origin of this abundant cell type remains unclear. Current hypotheses regarding glial evolution are mostly based on data from model bilaterians. Non-bilaterian animals have been largely overlooked in glial studies and have been subjected only to morphological analysis. Here, we provide a comprehensive overview of conservation of the bilateral gliogenic genetic repertoire of non-bilaterian phyla (*Cnidaria, Placozoa, Ctenophora*, and *Porifera*). We overview molecular and functional features of bilaterian glial cell types and discuss their possible evolutionary history. We then examine which glial features are present in non-bilaterians. Of these, cnidarians show the highest degree of gliogenic program conservation and may therefore be crucial to answer questions about glial evolution.

**Supplementary Information:**

The online version contains supplementary material available at 10.1186/s13064-024-00184-4.

## Background

**Fig. 1:**
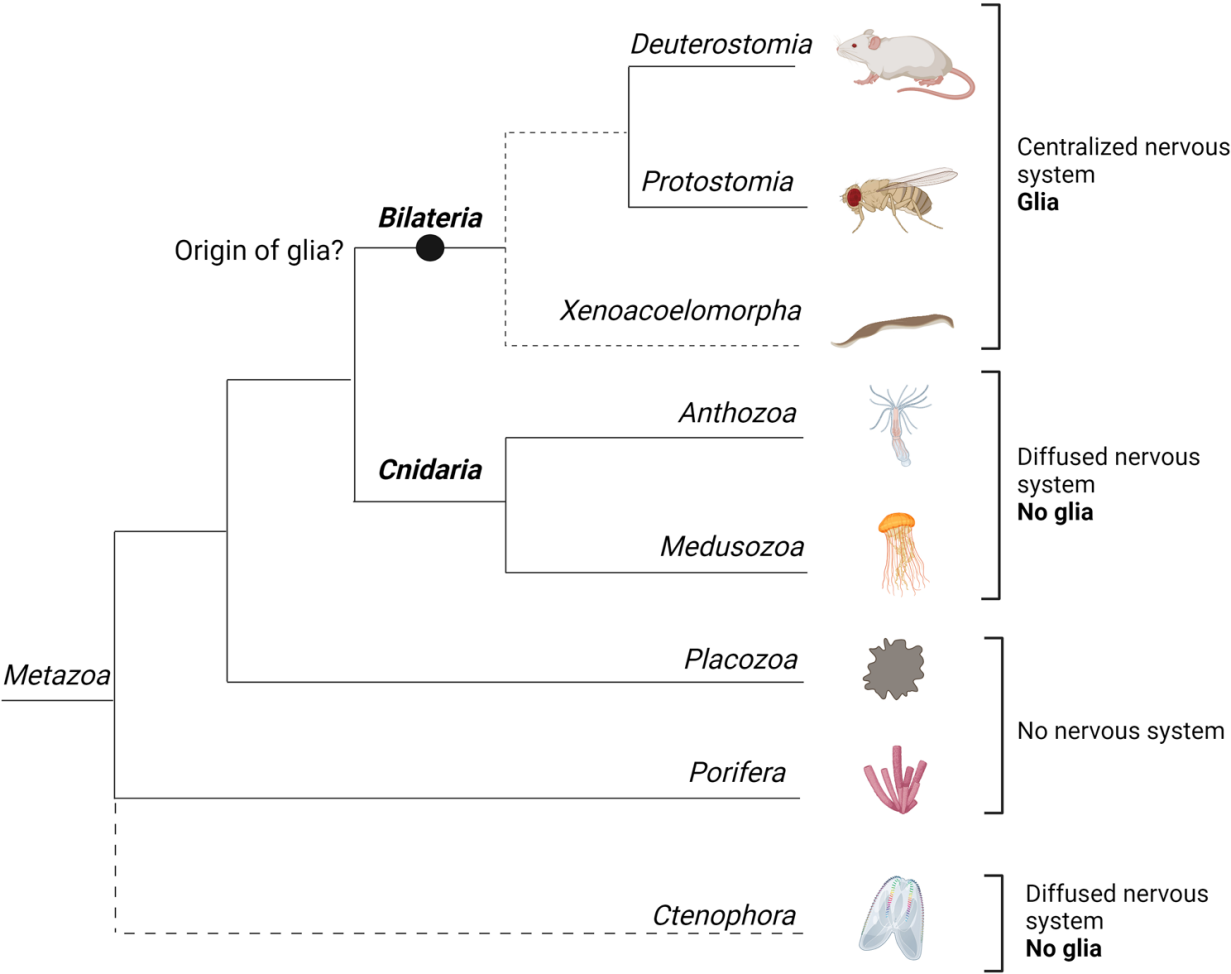
Phylogeny of *Metazoa* and distribution of glial genes. A metazoan phylogenetic tree of main animal groups includes the unresolved positions of *Ctenophora* and *Xenacoelomorpha* indicated by dotted branches [3]

Metazoans are broadly subdivided into bilaterians (*Protostomia* and *Deuterostomia*) and non-bilaterians (*Porifera* (sponges), *Placozoa*, *Ctenophora* (comb jellies), and *Cnidaria*). Nervous systems of bilaterian animals are generally composed of two cell types: neurons and glial cells. Among non-bilaterians only ctenophorans and cnidarians possess neurons forming a diffuse nervous system, but these phyla are believed to lack glia [1, 2] (Fig. 1).

Neurons are electrically active, which makes them the primary functional component of NSs. Although glia are considered supportive cells, they participate in almost every process in the nervous system of bilaterians, including neurotransmission, homeostasis, and also the important function of producing neurons [4, 5]. In the course of metazoan evolution, glial cells have acquired greater morphological and functional diversity [6]. This remarkable diversity is especially obvious in higher bilaterians such as vertebrates, as they include distinct types even within one glial cell class [7].

The emergence of glial cells is one of the key novelties in evolution of nervous systems. Important unanswered questions in glial biology are “Where did glial cells originate?”, and “What were their first functions?” Tracing origins of the first glia and identifying their original functions are therefore crucial to understand nervous system evolution.

Extensive studies of glia have mostly been confined to a few bilaterian model organisms, i.e., *Caenorhabditis elegans* (nematode) and *Drosophila melanogaster* (fruit fly) among invertebrates, *Danio rerio* (zebra fish), *Mus musculus* (mouse), *Rattus norvegicus* (rat), and *Homo sapiens* (human) among vertebrates. Some animals from other lineages, including non-bilaterians, have been screened for cells morphologically similar to glia. Glia-like cells have also been suggested in Xenacoelomorpha [2]. If this group of animals represents the earliest divergent bilaterian lineage, as several molecular phylogenetic analyses suggest, the emergence of glia-like cells may be traced back to the last common bilaterian ancestor. Overall glia are hypothesized to have evolved coincidentally with the appearance of the central nervous system (CNS), i.e., after the common bilaterian ancestor diverged from the *Cnidaria* [6, 8] (Fig. 1).

This view is being challenged by accumulating data collected from non-bilaterian animals. It was suggested that neurons and glia may have evolved at the same time, and that they have a common evolutionary origin [9]. This contrasts with the hypothesis that neuron-bearing, non-bilateral animals, *Ctenophora* and *Cnidaria*, have purely neuronal nervous systems [10]. Both frameworks assume that glial cells first emerged as metabolic support units for sensory neurons/organs, and that they then acquired an axonal support function, ensuring higher speed and more precise neuronal signaling by ensheathing axons. Finally, glial cells acquired other functions such as immune support.

Here we discuss genetic and cellular physiological features exhibited by bilaterian glial cells, and we also extend the investigation to common glial traits conserved in non-bilaterians that lack glial cells. In this review, we provide an overview of current knowledge about the extent to which the gliogenic genetic program in bilaterians is conserved and what function it serves in non-bilaterians. In the first part of the review, we provide a detailed examination of bilaterian glial cells, and their molecular signatures and functions. In the second part, we discuss conservation of the gliogenic program in non-bilaterians.

## Main text

### Morphology and physiology of bilaterian glial cells

Bilaterian glial cells are numerous and diverse, therefore defining them is challenging. Nevertheless, collectively, glia are considered non-neuronal cells of nervous systems. According to Shai Shaham [11], there are three primary characteristics of glial cells: morphology - glial cells are associated with neurons; physiology - glial cells do not conduct fast currents, do not possess neurotransmitter-laden vesicles and do not form presynaptic structures; origin (development) - glia, together with neurons, arise from neuroectoderm during embryogenesis.

Morphology is the most widely used means of identifying glia and is often the only method. Despite morphological variations observed in glial cells of different species (reviewed in [1]), close association with neurons is generally regarded as a common glia-specific feature. In addition, glial processes usually ensheath axons and creep through nerve bundles [12].

The astonishing morphological diversity of bilaterian glia seems related to diverse functions these cells perform: providing energy to neurons, maintaining the extracellular environment of neurons, immune response, serving as stem cells to generate glia and neurons in the adult brain, formation of the blood-brain barrier (BBB) [13–15]. On the other hand, the diversity of functions and glial cell types increases with the complexity of the nervous system. The increasing number of glia (from 10% of total brain volume in model invertebrates to over 50% in mammals), glia-to-neuron ratio, and glial cell complexity seems to support the idea that the earliest neurons did not need glial cells [6]. This led to a general definition of glia cells as “homeostatic cells of the nervous system” [6]. According to this definition, glial cells exist as housekeeping cells, whereas neurons serve as information processing units. At the same time, studies on *C. elegans* suggest that the first glia may have emerged at sensory receptive endings to control/support neuronal processing of incoming information about the environment [16]. Mammalian glial cells also assist in information processing by modulating synaptic activity and connectivity [17, 18]. Assigning a single function to glial cells in higher animals and using it as a universal diagnostic feature for all glial cells is therefore problematic.

Certain physiological features are helpful to consider when defining glial cells. Unlike neurons, glial cells are not known to generate action potentials. Nor has complete synaptic machinery been identified in glial cells. Other features tend to be glial cell type-specific, as it is possible to distinguish various glial cell types among model deuterostomes and protostomes.

As more molecular data are collected, classification of glia becomes more complex. In vertebrates, the term “glia” usually includes microglia and macroglia, i.e., radial glia, astrocytes, myelin-producing oligodendrocytes, and Schwann cells. There are several less numerous populations of glia, particularly in mammals, such as NG2 glia, known for their expression of a neurexin cell adhesion molecule NG2 and glial progenitor potential [19], pituicytes, tanycytes, and others residing in specific brain areas and resembling astrocytes transcriptionally [20–22]. In this review, we do not consider these glial subgroups separately.

In *Drosophila* seven types of glia have been identified [23], whereas *C. elegans* glia were divided into three groups (sheath glia, socket glia, and mesodermally derived glial cells) [16]. In other invertebrates, radial glia are often the only glial type reported [24]. On the other hand, more thorough examination of several non-model bilaterians reveals glial cells actively involved in neurotransmitter metabolism, which is an astrocytic feature [25]. Therefore, in order to trace glial origins, it is important to consider characteristics of different bilaterian glial types.

### Bilaterian glia cell types

#### Radial glia

Radial glia are neural stem cells and as such, are sometimes not considered strictly glial. They express stem cell markers such as Sox [15]. These cells give rise to neurons and astrocytes in addition to forming scaffolds used by newly generated neurons to travel to their final destination. Radial glia secrete Reissner’s fiber components and several markers generally considered specific to astrocytes, such as glutamate transporters and intermediate filament proteins, including glial fibrillary acidic protein (Gfap) and Nestin [26]. Radial glia are elongated cells, and extend long processes through the neuropil. They are the earliest type of glia to develop in vertebrates, but are sparse in adult mammalian brains. Radial glia have been reported in both deuterostomes (*Vertebrata*, *Echinodermata*, *Hemichordata*) and protostomes (*Annelida*, *Arthropoda*) [27, 28]. They are the only type of glia identified so far in *Echinodermata* [24], and they perform a phagocytic function in addition to their neurogenic and scaffolding functions [29]. Given that radial glial cells are present throughout the *Bilateria*, that they can generate both neurons and glial cells, and are the first neural cell type to develop, they may have been the first glia to emerge in animals with nervous systems.

#### Astrocytes/astrocyte-like

**Fig. 2:**
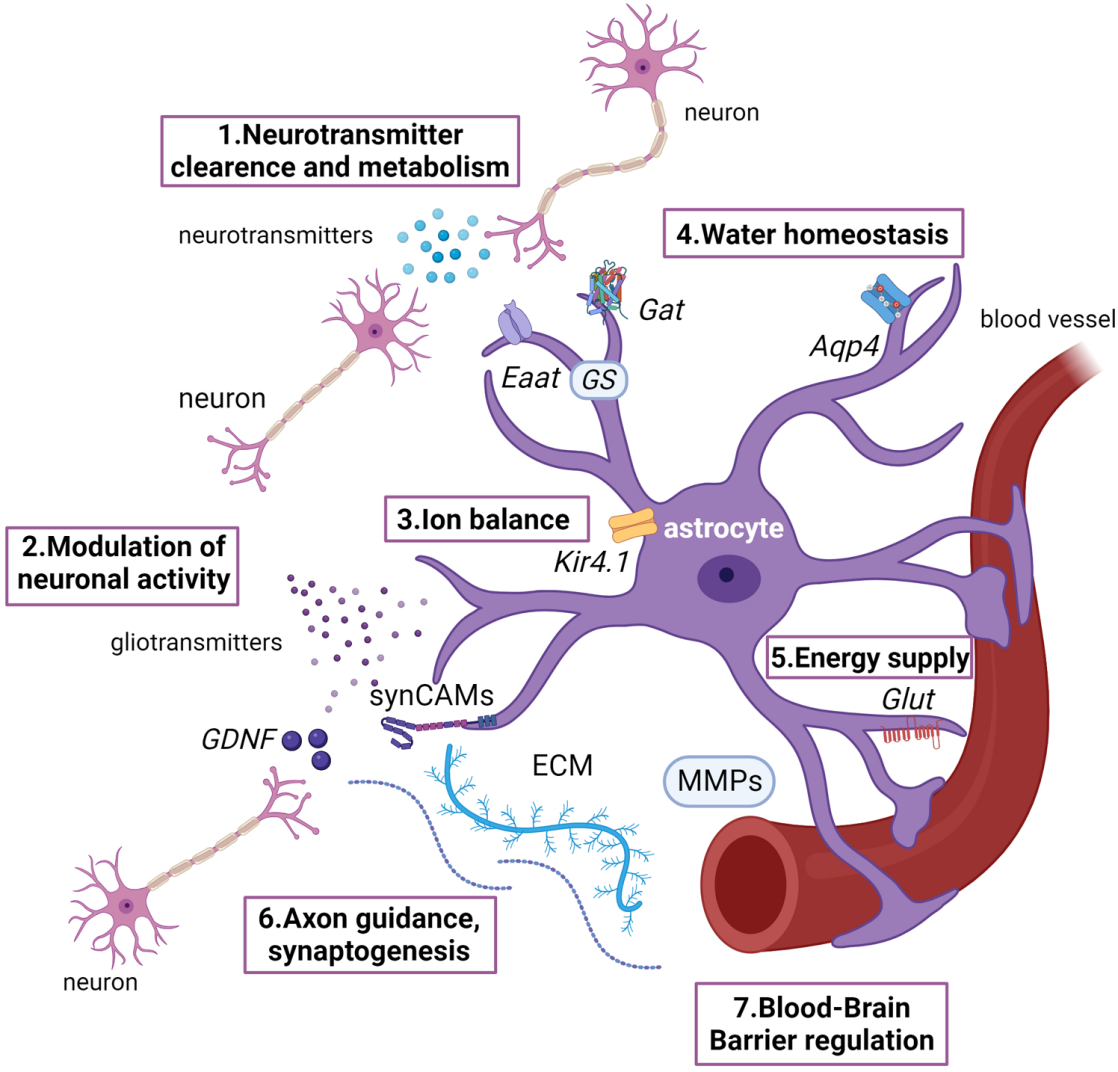
Astrocyte functional diversity. Each function is fulfilled by astrocyte-expressed, secreted proteins

Astrocytes are glial cells in the most classical sense. They are closely associated with neurons, and are the primary cells maintaining homeostasis of vertebrate CNS. Astroglia fulfill many functions in the nervous system such as regulating ionic and neurotransmitter composition of the neuronal environment [30, 31]; maintaining water homeostasis [32]; energetic support of neurons [33]; maintaining the blood-brain barrier [14, 34]; synaptogenesis [35], axon guidance [36]; and phagocytosis [37, 38] (Fig. 2). Each of these functions is evidenced by expression of genes such as ion channels, including potassium channels (Kir4.1) [39, 40], GABA and glutamate transporters, glutamine synthetase (Gat, Eaat, Gs) [41, 42]; aquaporin channels (Aqp4) [43]; glucose transporters (Glut) [44]; BBB-regulating factors such as matrix metalloproteinases (MMPs) and glial-derived neurotrophic factor (GDNF) [14]; ECM proteins that are synaptogenic, e.g., thrombospondins, and axon guiding, e.g., SynCAMs factors [45, 46]; cell death abnormality (Ced) pathway components involved in engulfment and phagocytosis [47] (Fig. 2). A metabolic enzyme, Aldh1l1 (aldehyde dehydrogenase 1 family member L1), and intracellular signaling Rab6 are pan-astrocytic markers in rodents [47, 48]. Some of these markers, especially those involved in glutamate metabolism, have been successfully used to identify glial presence in invertebrate bilaterians, e.g., planarians [25]. Several transcription factors (TFs) including *Nfe2l1*(bZIP TF) regulated by *Sox9* [49], *Klf15* (Kruppel-like family), and *Scl* (bHLH TF) [50] are involved in astrocyte development. Astrocytes demonstrate “classical” glial morphology: multiple processes extending into the neuropil, surrounding synapses, and neuronal processes. Based on these criteria in addition to expression of Gfap, astrocyte-like glia were identified in early-branching bilaterians - acoels [51, 52]. Glial cells that morphologically and functionally resemble astrocytes are also found in zebrafish (deuterostomes), *C .elegans*, and *Drosophila* (protostomes). Because astrocyte-like glial cells are present throughout bilaterian lineages, astroglia may have been the first true glia type to evolve.

#### Oligodendrocytes, Schwann cells, wrapping glia

**Fig. 3:**
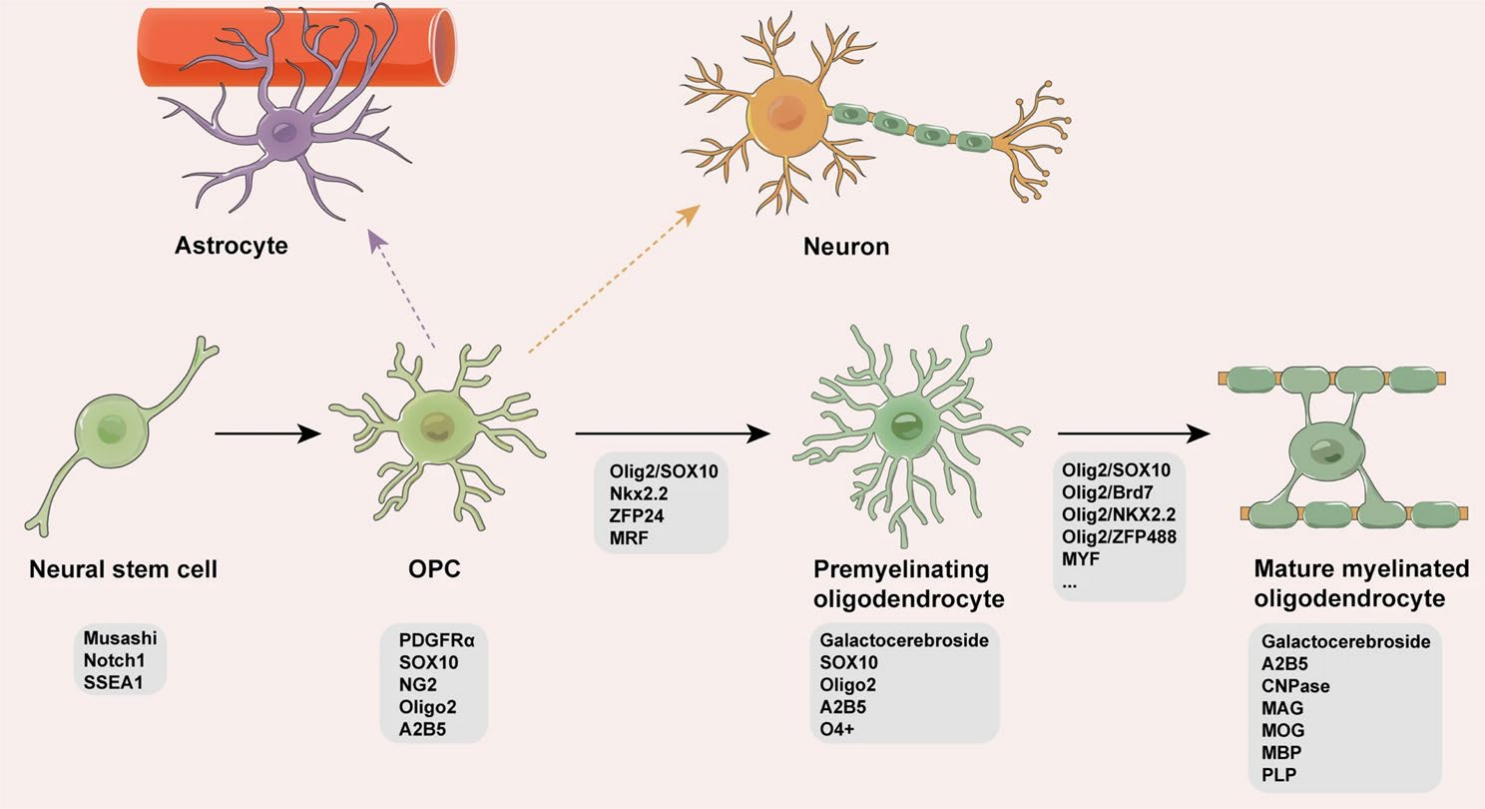
Developmental program of mammalian oligodendrocytes. Neural stem cells give rise to oligodendrocyte progenitor cells (OPCs), which begin to express oligodendrocyte markers (*Sox10*, *PDGFRa*). OPCs potentially differentiate into astrocytes, neurons, and oligodendrocytes, although the former two signaling pathways are under debate. *Olig2* and *Sox10* genes, among others, drive oligodendrocyte maturation. Myelin constituents, such as MBP (myelin basic protein) and PLP (proteolipid protein), are expressed by mature myelinated oligodendrocytes. Image taken from [59]

Myelin production is the major function of oligodendrocytes in the CNS and Schwann cells in the PNS of vertebrates. The appearance of myelin correlates with jaw development, which suggests that it is a recent invention, from an evolutionary point of view [2]. Myelin-associated proteins are used as oligodendrocyte and Schwann cell markers [53, 54]. At the same time, oligodendrocyte lineage markers are present in species that do not possess myelin. These include TFs that drive oligodendrogenesis such as *Sox10* and *Olig2*, as well as a well-known marker of oligodendrocyte progenitors - platelet-derived growth factor receptor (PDGFRα) [55] (Fig. 3). This may indicate a common gliogenic program dating back to invertebrates. Even among vertebrates, not all oligodendrocytes produce myelin. The common feature that oligodendrocytes do share, however, is axonal ensheathment. Glial cells covering axons with their membranes are found in various animals, including protostomes [2]. Ensheathment of axons is hypothesized to have evolved to allow increased conduction speed and precision of neuronal signaling by blocking electrical crosstalk between axons [9, 56]. Simultaneously, these cells may have provided nutrients for neurons. Oligodendrocytes share the function of providing metabolic support to neurons with astrocytes and express the same glutamate and GABA transporters [57]. Oligodendrocytes and astrocytes form networks via gap junctions, contributing to ionic buffering during neuronal activity [58]. Therefore, distinguishing between oligodendrocytes and astrocytes may be trivial only in vertebrates, as functions of both these cell types are performed by the same cells in animals with simpler nervous systems.

When considering evolutionary roots of glia, it is helpful to consider the gliogenic vs. neurogenic program. It is generally believed that oligodendrocytes arise from the same progenitors as neurons [60]. If this is the case, only the astrogenic program should be searched for in organisms with simple nervous systems, in order to reveal glial evolution. However, some progenitors produce both oligodendrocytes and astrocytes [61]. Radial glia produce all three cell types, depending on the developmental stage [62]. Oligodendrocyte progenitor cells (OPCs) may also generate all three cell types (Fig. 3). Therefore, it is rather difficult to identify a strictly gliogenic program.

#### Microglia

**Fig. 4:**
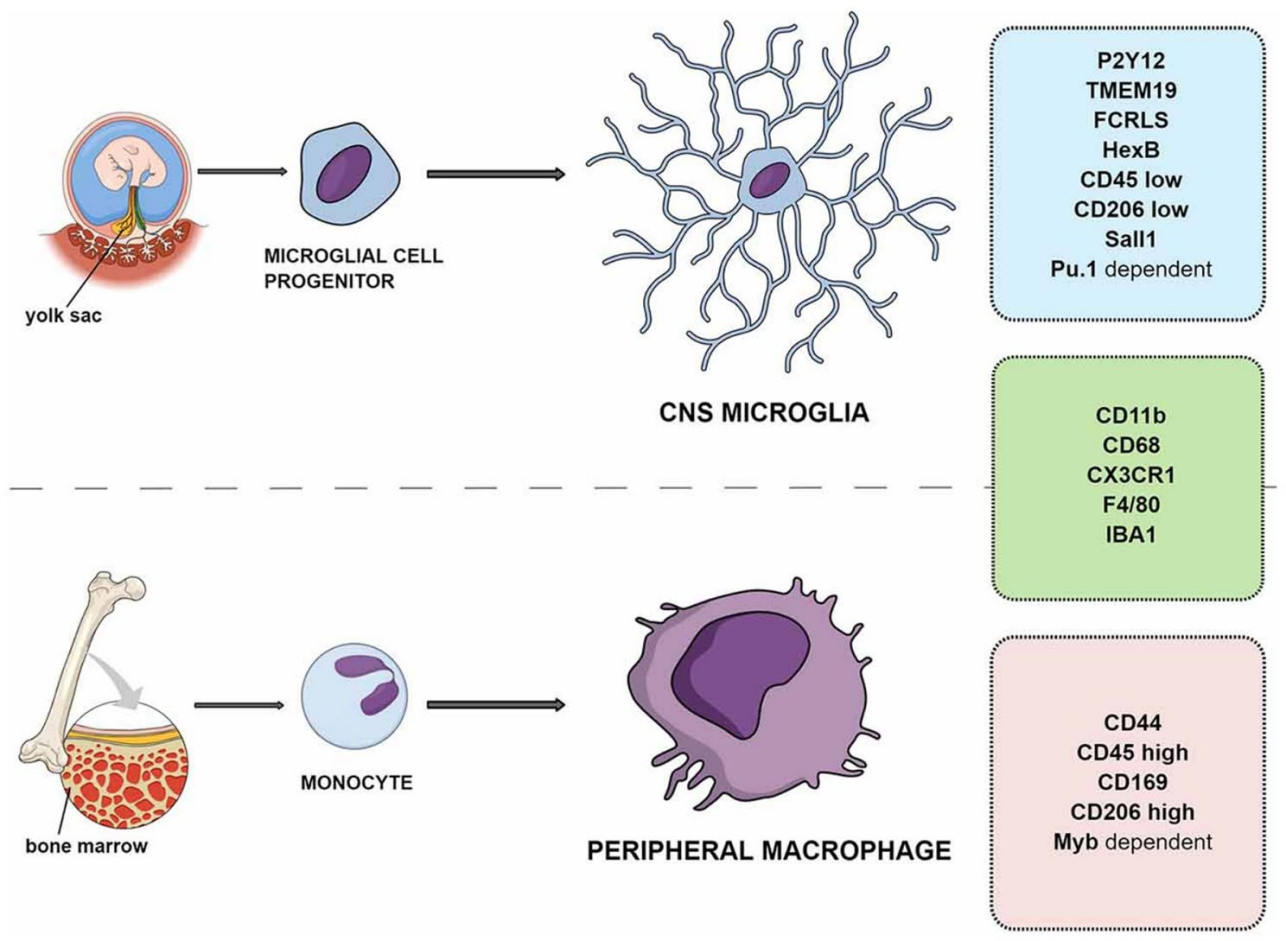
Mammalian microglia and peripheral macrophage ontogeny and markers. The two cell types share a majority of markers, although their origins differ. Image taken from [73]

Unlike macroglia, which originate within the ectoderm, vertebrate microglia have a mesodermal origin. Similarly, *C. elegans* possesses 6 mesodermally derived glial-like cells in the nerve ring (GLRs) serving as connections between neurons and muscles [16]. The function of GLRs is not clear, but they engulf dead cells [63]. In mammals, microglia serve as resident macrophages of the CNS [64]. Because one of the main functions of microglia is debris clearance and degradation, markers of phagocytic pathways such as P2ry12 and lysosomal enzymes such as Hesb are abundantly expressed by these cells [47, 65]. To this end, microglia and astrocytes share the function of synapse pruning and engulfment [66]. Similarly, both cell types react to injury and inflammation. Even more so, astrocytes assume the role of phagocytes in case of microglial dysfunction [67]. Like other neural cells, vertebrate microglia express various ion channels, neurotransmitter receptors, and transporters [68]. In general, however, the transcriptome of microglia differs significantly from that of macroglia in that it is enriched with markers related to immune system processes and macrophages, e.g. CD45, CD68, but also microglia-specific markers, e.g. TMEM119 [65, 69] (Fig. 4). The transcriptional program driving microglial identity is drastically different from that of macroglia [70]. *Spi1* (or *Pu.1* - *Ets*-domain TF) and *Irf8* (interferon regulatory factor family) are the main TFs driving microgliogenesis in various vertebrate species [71]. Morphologically, resting microglia resemble astroglia in that cells extend processes from the central soma. Upon activation in response to injury microglia change their morphology and aggregate at the lesion site. The complexity of the ramified structure of microglia varies with the overall trend of increasing in evolution. However, unlike astrocytes, human microglia do not display the most complex morphology compared to other vertebrates [71]. Apart from vertebrates, insects (*Arthropoda*), leech (*Annelida*), and molluscs are reported to have microglial cells [2]. Among these, leech microglia have been studied most thoroughly, albeit using few molecular markers [72]. Surprisingly, no other glial cell types have been identified in leeches. It is not clear to what extent the vertebrate microglial molecular program and functions are conserved in invertebrates, due to the paucity of studies.

**Fig. 5:**
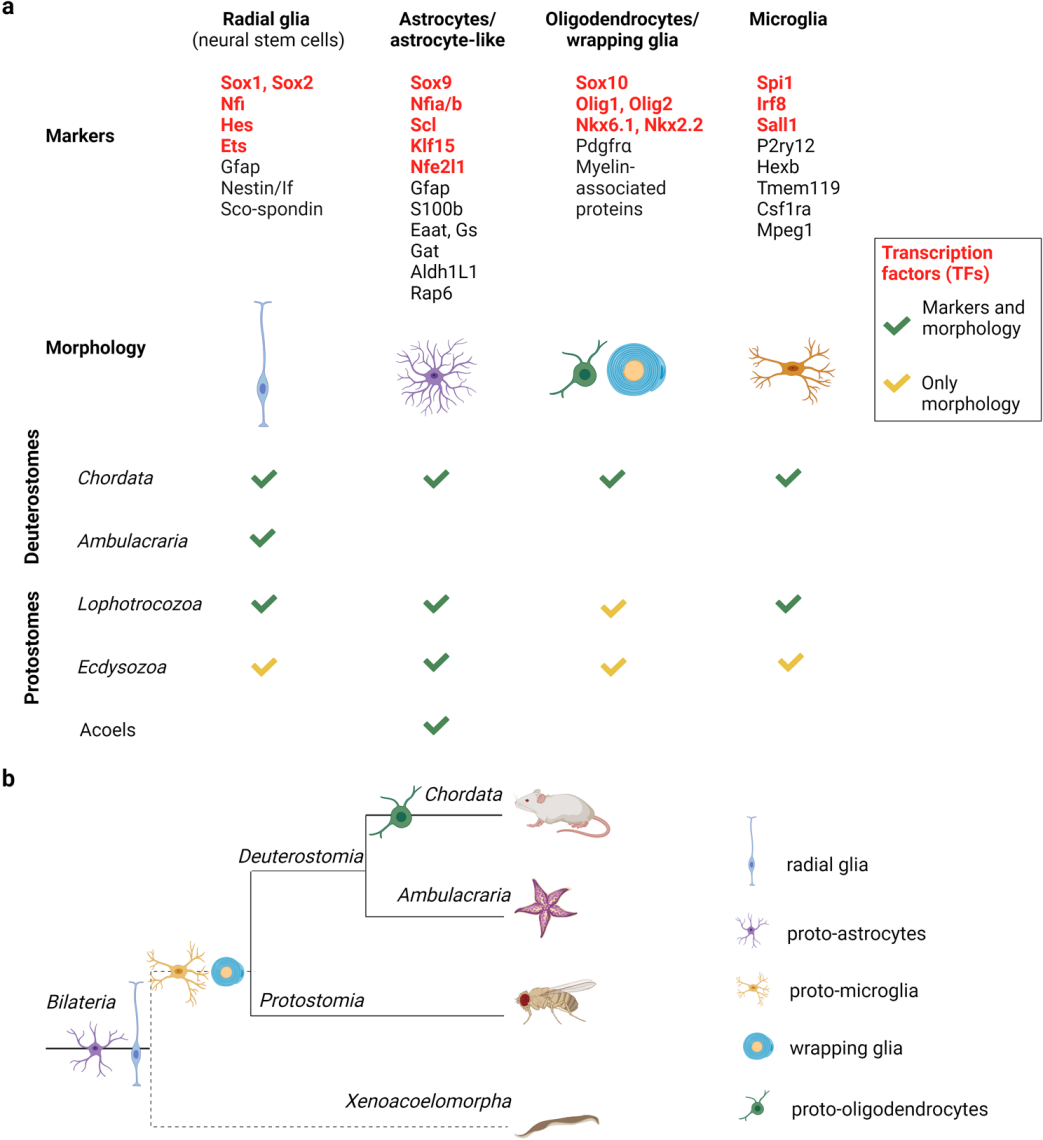
Bilaterian glial cell types: molecular markers, morphology, and evolutionary processes. (**a**) Glial cell type conservation in bilaterian phyla. Molecular markers and morphological features of major glial cell types are shown. (**b**) Evolutionary tree of glial cell types based on available bilaterian studies

Figure 5 summarizes glial cells and their marker genes found in major model animals. With the emergence of molecular identifiers for each glial cell type, it has become possible to identify various glial cells in bilaterian lineages other than vertebrates and insects (Fig. 5a). Nevertheless, few glial markers have been searched for in non-model organisms. Based on data obtained mostly from histological studies, it is likely that radial glia were the first glial type to evolve. These cells combine features of both neurons and glia and give rise to both. The first true glia to emerge may have been astrocyte/oligodendrocyte-like cells that assumed several key functions, including metabolic support for and electrical insulation of neurons. Glial cells reminiscent of microglia can be found in basal bilaterians, but were likely to have emerged later in evolution (Fig. 5b).

### Phylogenetic distribution of glia-related genes

**Fig. 6:**
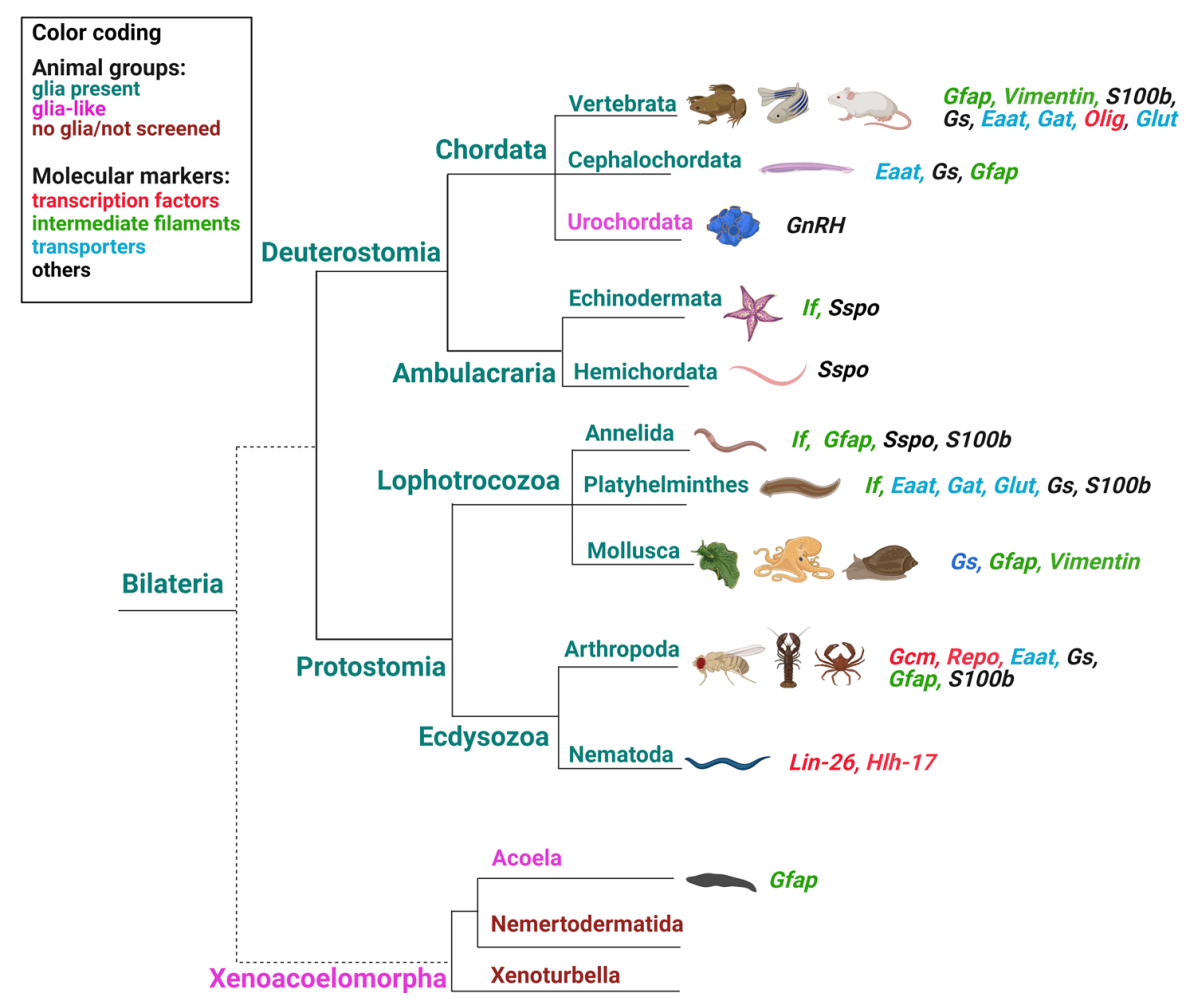
Phylogenetic distribution of major glial markers. Presence of glia is inferred from reports on cells morphologically and genetically similar to well-developed glia of vertebrates. Glial markers identified in each phylum are specified and color-coded. Representative species screened for glial markers are enumerated in Supplementary Table S1 and accompanied by literature references

Molecular markers have long been used to identify cell types and to identify their functional characteristics. These have become even more prevalent with the emergence of omics techniques. The most widely used glial marker is an intermediate filament (If) protein, Gfap, which was first identified in vertebrates. Gfap immunostaining has been used to identify potential glia in various invertebrates, including an early-branching bilaterian, *Symsagittifera roscoffensis* (*Xenacoela*) [51] (Fig. 6). Immunostaining with GFAP antibodies documented the distribution of putative glial cells; however, *Gfap* labels only a subset of astroglia in vertebrates [47], and is also expressed in non-glial cells [74]. In addition, *Gfap* is not expressed in other glial types, like oligodendrocytes [47]. Intermediate filaments including *Gfap* are prominent features of glial cells in several bilaterians [75] (Fig. 6). Apart from *Gfap*, another intermediate filament protein, vimentin, is expressed in glial cells in the snail *Megalobulimus abbreviatus* [76]. In mammalians, *vimentin* is expressed in radial glia and astrocytes during early differentiation stages, and is later replaced by *Gfap* [77]. Overall, it is less glia-specific than *Gfap*, even in invertebrates [78]. Interestingly, intermediate filament-1 protein (If-1) is expressed in planarian glia [25].

Other vertebrate glial markers searched for and used to identify glia in invertebrates include glutamine synthetase (*Gs*) in the lobster (*Panulirus argus*) [79] and *Aplysia* [80], S100 calcium-binding protein B (*S100b*) in the giant prawn (*Macrobrachium rosenbergi)* [81] and flatworm (*Christensenella minuta*) [82], transporters for glutamate (*Eaat*), GABA (*Gat*), and glucose (*Glut*) in planaria (*Schmidtea mediterranea*) [25] (Fig. 6). Expression of *Eaat*, *Gs*, *Gfap*/*vimentin*/*If* genes was explored in the lancelet, documenting glia in the *Cephalochordata* [83]. Sco-spondin (*Sspo*), an extracellular matrix (ECM) glycoprotein, the main component of the Reissner’s fiber [84], is another glial marker. It is secreted by radial glia and was used to identify glia in both deuterostomes and protostomes [27, 85]. Some glial markers seem phylum/class-specific and have been used to identify glia in certain animals. For example, gonadotropin-releasing hormone (GnRH), is expressed in glia of a urochordate, *Ciona intestinalis* [86], while *Aplysia* glia secrete a protein called Ag [87] (Fig. 6).

### Gliogenic program in bilaterians

Expression of glial molecular markers, i.e., functional genes, is driven by TFs and extracellular cues. Therefore, by unraveling the genetic developmental program driving gliogenesis, it may be possible not only to define glia more precisely, but also to trace back their evolutionary origins. In *Drosophila*, it has been possible to identify TFs that drive expression of glial-specific markers, the so-called ‘binary switches’, such as *Gcm and Repo* [88, 89], demonstrating that invertebrates have a more robust intrinsic system for neuro/gliogenesis than had been thought previously. The vertebrate situation is more complex, particularly among mammals. No specific TFs responsible for glial cell fate acquisition have been identified per se, even though some, such as *bHLH*s, *Sox* group E, *Ets* and *NfI* family, are obviously involved [49, 90, 91]. Therefore, gliogenic programs in protostomes (insects) and deuterostomes (vertebrates) do not appear to be homologous.

#### Glial cells missing (Gcm)

*Gcm* is a master regulator of gliogenesis in *Drosophila* [92–94]. *Gcm* is indispensable for gliogenesis: a mutation in this gene turns presumptive glia into neurons [92]. All lateral glia of *Drosophila* rely on *Gcm* for their development, but specific subtypes of glial cells are established via different mechanisms [23]. *Drosophila* has two *Gcm* genes with partially redundant functions [95, 96]. *Gcm2* seems less important for glial differentiation, but is involved in macrophage development [95, 96]. Both *Gcm* genes promote postembryonic neurogenesis, in addition to glial development in *Drosophila* CNS [97]. The neuro/gliogenic functions of *Gcm* homologs have also been shown in other taxonomic classes. *Gcm* neural expression and function has been demonstrated in crayfish (crustaceans) [98]. It is not clear whether crustacean *Gcm*-expressing cells are glial. Nevertheless, as in vinegar flies, crustacean *Gcm* seems to be co-expressed with *Repo*, which is an important gliogenic TF under *Gcm* regulation in *Drosophila*. Sea urchins (*Echinodermata*) also possess *Gcm*, although it is not expressed in neural tissue [99]. It functions in pigment cell specification instead [100]. Importantly, however, in both *Drosophila* and *Strongylocentrotus purpuratus*, *Gcm* expression is driven by *Notch* [99, 101]. In bilaterians, *Notch-Delta* signaling promotes gliogenesis [102, 103] by activating glial genes and sustaining neuronal precursors in both vertebrates and invertebrates [104–106]. *Hes/hairy* are primary downstream targets of *Notch*, inhibiting neurogenic bHLH TFs such as *Atonal* and *Achaete–Scute complex* [102, 106, 107]. In *Drosophila*, Notch drives *Gcm* expression to give rise to subperineurial glia (SPG) [101]. However, Notch has an opposite effect on *Gcm* in the sensory organ precursor lineage of the peripheral nervous system (PNS) [108]. Likewise, there is no unified effect of *Notch* on glial differentiation in vertebrates, where *Notch* promotes certain glial types such as astrocytes and radial glia, but not oligodendrocytes (reviewed in [109]). Functions of *Gcm* homologs in vertebrates are still debated. *Gcma* (or *Gcm1*) and *Gcmb* (or *Gcm2*) have been isolated in mammals [110]. These are expressed predominantly in tissues other than neural tissue: *Ggma* is expressed in placenta [111, 112], *Gcmb* in parathyroid glands [113]. However, both genes induce generation of neural stem cells [114]. The neurogenic role of *Gcma* is conserved in chickens [114, 115]. Moreover, rodent *Gcma* induces gliogenesis and drives astrocyte differentiation [116, 117]. In zebrafish, one *Gcm* gene was isolated and named *Gcmb*, due to its similarity to mammalian *Gcmb.* It is expressed in macrophages and contributes to pharyngeal cartilage formation [118]. In *C. elegans*, all glial cells express the Zn-finger transcription factor, *Lin-26* [119]. Epithelial cells also express *Lin-26* and are transformed into neurons in *Lin-26* mutants, highlighting the conserved linear relationships between glia, neurons, and epithelia. The function of *Lin-26* is reminiscent of the function of *Gcm*. However, Lin-26 and Gcm proteins are not clearly homologous. A definite role for Lin-26-related genes in gliogenesis is currently unknown except in *C. elegans*, suggesting that this mechanism may have been acquired independently in that lineage. In bilaterians that have only a single *Gcm* gene, its neural and gliogenic functions are not yet clear. In lineages that have two *Gcm* genes, homologs of *Drosophila Gcm1* show gliogenic functions. In both protostomes and deuterostomes, *Gcm* is tightly regulated by Notch [101, 114, 120]. Thus, the Notch-regulated *Gcm* program seems conserved in bilaterians.

#### Reversed polarity (Repo)

*Repo* is a homeobox gene downstream from *Gcm* in *Drosophila*, which ensures terminal differentiation of glial cells [121]. *Repo*, in turn, drives expression of glial-specific markers, including *Pointed*, which is also regulated by *Hairy* [88, 122]. In addition to *Pointed, Tramtrack69*, *Loco*, and *M84* are effectors of *Repo*, which participate in glial differentiation and morphogenesis in *Drosophila* [88]. *Repo* mutants demonstrate reduced cell number and poorly differentiated glia [123]. *Repo* is not known to have a gliogenic role in animals other than in insects. Vertebrates lack the gene for Repo altogether.

#### Oligodendrocyte transcription factor (Olig)

*Olig* belongs to group A bHLH genes. *Olig1* and *Olig2* promote oligodendrocyte differentiation in mammals [124, 125]. *Olig* genes are among several TFs that couple neuronal and oligodendrocyte specification [60, 126]. In addition, a subpopulation of astrocytes expresses *Olig2* [127]. These findings indicate that Olig TFs are not specific oligodendrocyte genes. The primary function of oligodendrocytes is to myelinate neurons to ensure fast action potential propagation. Myelin is a new invention, from an evolutionary point of view, as it is associated only with vertebrates [2, 128]. On the other hand, axonal ensheathment by glial membranes is observed in invertebrates as well. This suggests that the first functions of glia may have been metabolic support for neurons. Interestingly, *C. elegans* possesses a bHLH gene, *Hlh-17*, and regulation of its expression is similar to that of mammalian *Olig2* [129]. *C. elegans* single-cell data confirm its mostly glial expression, although it is also expressed in neurons [130]. Therefore, *Olig-like* genes may have emerged as TFs with glio/neurogenic functions in the common ancestor of deuterostomes and protostomes.

#### Hairy and Enhancer of split (Hes)

*Hes* are involved in many developmental processes, including suppression of proneural bHLH genes, and promotion of gliogenesis [131, 132]. Mammalian *Hes* genes are homologs of *Drosophila Hairy* and *Enhancer of Split*. Vertebrate Hes1, Hes3, and Hes5 particularly promote gliogenesis at a later stage of the developing brain, and control production of neural stem cells at an earlier neurogenic stage [133]. Interestingly, *Hes5* is specifically expressed in mammalian Muller glial cells [134]. Its expression is regulated by *Gcm* genes at an early stage to induce neural stem cell generation, and it is later replaced by activation by *Notch* [114]. *Hes* genes are known effectors of *Notch* in mammals, as well as *Drosophila* [133, 135, 136]. *Gcm, Hes*, and *Notch* could therefore be important for gliogenesis in various lineages, even though *Gcm-Notch* synergy driving glia generation in *Drosophila* seems independent of *Hes* [137].

#### Nuclear factor I (NfI)

*NfI* genes are CCAAT box element-binding TFs [138]. In vertebrates, the *NfI* family is composed of four genes: *NfIa, NfIb, NfIc* and *NfIx*. These are important for development of various tissues, including the nervous system [138, 139]. *NfIa, NfIb*, and *NfIc* promote differentiation of radial glial cells into both glia and neurons [140]. *NfIa* directly induces expression of glial-specific genes [141], and initiates gliogenesis under the control of *Sox9* [142]. *Notch* induces *NfIa* to drive gliogenesis via *Hes* genes [141]. A single NfI gene is present in *Amphioxus*, *C. elegans*, and *Drosophila* [138, 143, 144]^]^, but in these animals, NfI does not appear to have a gliogenic function.

#### SRY-box transcription factor (Sox)

*SoxE* is a group of genes belonging to a high mobility group (HMG)-box *Sox* family, which serves various functions, including nervous system development. In mammals, *SoxE* genes including *Sox8, Sox9*, and *Sox10* are essential for glia generation [145], among which, *Sox9* is a major neuron-glia switch, as it directly regulates expression of *NfIa*, is indispensable for astrogenesis, and prevents neurogenesis [90, 91, 142]. *Sox8* and *Sox10* contribute to oligodendrogenesis [145]. *Sox10* is induced by *Olig2* and interacts with *Olig1*, driving expression of myelin genes and suppressing expression of astrocyte-related genes [91]. *SoxE* genes also have gliogenic functions in jawless vertebrates (lampreys) [55]. Even though these animals lack oligodendrocytes, they possess the genetic regulatory network required for oligodendrogenesis, including *SoxE* and *Olig* orthologs. Gliogenic functions of *SoxE* genes have not been reported in protostomes. In crustaceans, *SoxE* orthologs regulate gonad and embryo development [146, 147]. In *Drosophila* an ortholog of vertebrate *Sox8,9,10* is expressed in the gut and gonads, and is required for intestinal epithelium function [148, 149]. The gliogenic function of class E *Sox* genes seems specific only in chordates.

#### Erythroblast transformation specific (Ets) family

*Ets* proteins belonging to the group A bHLH TF family, regulate various developmental processes, including glial cell differentiation. In *Drosophila*, an *Ets* TF, *Pointed* (*Pnt*), is activated by *Gcm* via *Repo* to induce expression of glial markers in several glial cell types [88, 150, 151]. Vertebrate homologs of *Pnt, Ets-1* and *Ets-2*, drive radial glia formation in *Xenopus* [152]. In mammals, two *Ets* family TFs (*Ets1* and *Fli1*) are expressed mainly in astrocytes and oligodendrocytes [153]. Other *Ets* family members drive gliogenesis in rodent peripheral nervous systems and promote oligodendrocyte proliferation [154, 155]. Therefore, the gliogenic nature of *Ets* is common to both protostomes and deuterostomes, necessitating further investigation regarding its conserved function in non-bilaterians.

Extracellular signaling pathways regulating gliogenesis: In addition to intrinsic factors, extracellular cues influence cell fate acquisition. Various signaling pathways have been implied in bilaterian gliogenesis [156]. As already mentioned, Notch-Delta signaling is a major, versatile pathway in bilaterian gliogenesis [102, 103]. The Notch function of maintaining a pool of stem cells in the nervous system in the form of either glio-neuro-precursors or mature glial cells seems conserved among bilaterians. Other signaling pathways driving gliogenesis in vertebrates include JAK-STAT [157–159], which talks with Notch-Delta to drive glial differentiation [160], BMP signaling [161, 162], and Hedgehog [25, 156]. Control of glial cell differentiation by these signal pathways is well known in vertebrates. Considering the presence of these signal genes in early-branching animals, the function of these signals in pre-bilateral animals should be an interesting subject of study.

### Repertoires of “gliogenic” genes in non-bilaterians

Extant lineages of non-bilaterian animals include *Placozoa*, *Porifera* (sponges), *Ctenophora* (comb jellies), and *Cnidaria*, of which *Ctenophora* and *Cnidaria* possess nervous systems (Fig. 1). While *Cnidaria* is clearly the closest sister group to *Bilateria*, phylogenetic relationships among other non-bilaterians are still debated [3, 163, 164]. Recently, these phyla have been actively studied, particularly in the context of nervous system evolution. Thanks to advancements in sequencing and molecular techniques, it has been possible to address questions regarding nervous systems of ancestral metazoans. Prior to investigating gliogenic program conservation in non-bilaterians, it is necessary to characterize nervous systems of these animals, as discussed below.

### Nervous system features of non-bilaterians

Placozoans and poriferans do not possess neurons, but their genomes contain pro-neural TFs and encode proteins required for synapse formation and neurotransmitter synthesis [165, 166]. Therefore, studies focused on unraveling functions of neuro-associated genes in these animals are expected to shed light on evolution of the nervous system. In addition, nerveless *Placozoa* and *Porifera* display epithelial contractile responses to neurotransmitters, such as short peptides, glutamate, GABA, and glycine [167–170]. The first nervous system probably heavily relied on peptidergic signaling, as evidenced by an extensive repertoire of neuropeptides in non-bilaterians with nervous systems, cnidarians and ctenophores [171, 172]. *Cnidaria* and *Ctenophora* both possess neurons organized into nerve nets with regional compartmentalization (Table 1). Phylogenomic analyses place *Cnidaria* as a sister group to all *Bilateria*, whereas *Ctenophora* could be one of the earliest-branching lineages of *Metazoa*. Recent molecular and structural studies on *Ctenophora* have revealed that they have, at least in part, unique neural characteristics, acquired independently from other metazoans [173, 174]. On the other hand, conserved neurogenic TFs (SoxB, bHLH) and vesicle secretion exist in *Bilateria, Cnidaria* and *Ctenophora*. Although it is still unclear whether chemical neurotransmitters are recruited in ctenophoran nervous systems. Current evidence suggests that glutamate and glycine, but not GABA, are involved in muscle contraction [175, 176]. Unlike ctenophores, cnidarians share all key features of bilaterian nervous systems, including a diverse repertoire of gene orthologs involved in neurogenesis and neural functions [177–179]. Cnidarian nervous systems generally consist of a nerve net and regional condensations in the oral (“nerve ring”) and aboral regions. Although cnidarian nervous systems are rich in neuropeptides, classical chemical neurotransmitters such as nitric oxide (NO), glutamate, GABA, glycine are also involved in neural functions (Table 1). Small transmitters, including glutamate, can perform both non-neuronal and neuronal functions, as may be the case in *Cnidaria*. Regardless, glutamate and glycine may have been recruited by neurons as neurotransmitters at some point in evolution [175]. More functional studies are required to understand whether this occurred with emergence of the first neurons, however. It is also debated whether acetylcholine and monoamines function as neurotransmitters in cnidarians, since a complete gene set of canonical pathways for synthesis of these molecules is absent [175, 180].

**Table 1:**
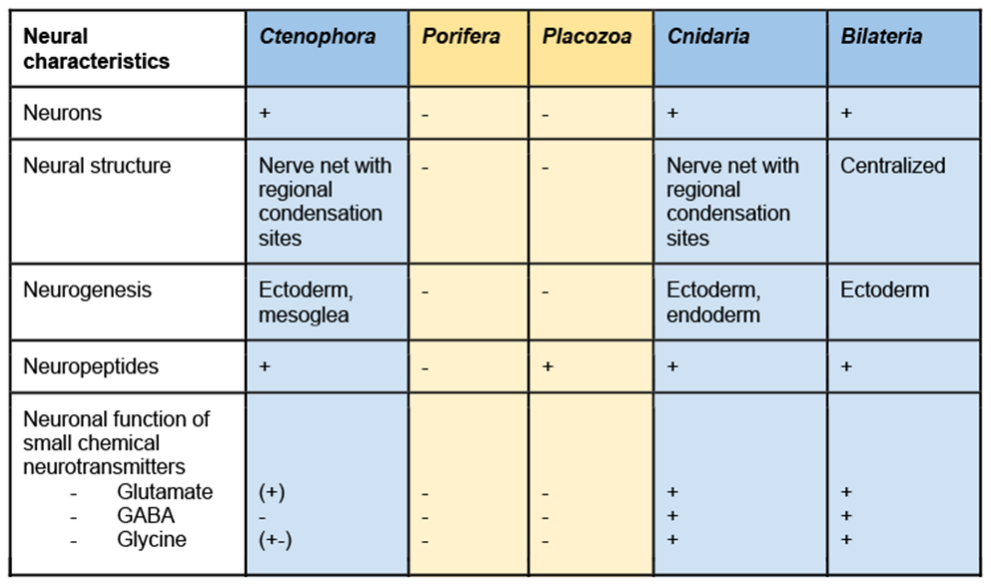
Neuronal features in non-bilaterians. Nerveless phyla are highlighted in yellow. Phyla possessing neurons are highlighted in blue

In summary, neuronal genes and modules can be found even in non-bilaterians without nervous systems, as described above. This suggests that many “neural” genes already existed before emergence of the nervous system, and that they acquired neural functions with emergence of the nervous system. Similarly, glial genes and modules may be present in lineages without glia. An interesting matter is to what extent the bilaterian gliogenic program is conserved in non-bilaterians. Glial and neuronal developmental programs are tightly intertwined, which complicates identification of strictly glial genes. Nevertheless, as discussed, several specific glial TFs and effector genes are present throughout *Bilateria*.

In this part of the review, we used comparative sequence analysis and the literature to survey orthologs of bilaterian glial TFs in non-bilaterians. It is common not to find specific orthologs for bilaterian genes in early-branching lineages. Orthologous family members should be analyzed instead. Functions of these non-bilaterian TFs can be assumed from their sequence similarities to bilaterian orthologs, but expression patterns and effector genes must be considered for accurate functional assessment. Therefore, in addition to sequence similarities, we explored expression patterns of some of these glial TF orthologs.

### Glial transcription factors and signaling pathways in non-bilaterians

#### NfI

TFs belonging to the *NfI* family have a gliogenic function in vertebrates. As discussed before, a single member of the *NfI* family is present in invertebrates and does not seem to contribute to gliogenesis. Similarly, in the cnidarian, *Nematostella vectensis*, a single *NfIx*-like gene was identified (Table 2). It is expressed in the central region at blastula stage and is thought to be involved in endomesoderm specification [181]. It is unclear if the *NfIx*-like gene in *Nematostella* is expressed beyond this developmental stage and whether it has other functions. A single *NfI-like* gene was also identified in *Porifera* and *Placozoa* (Table 2). To date, no expression pattern of this gene has been reported.

**Table 2:**
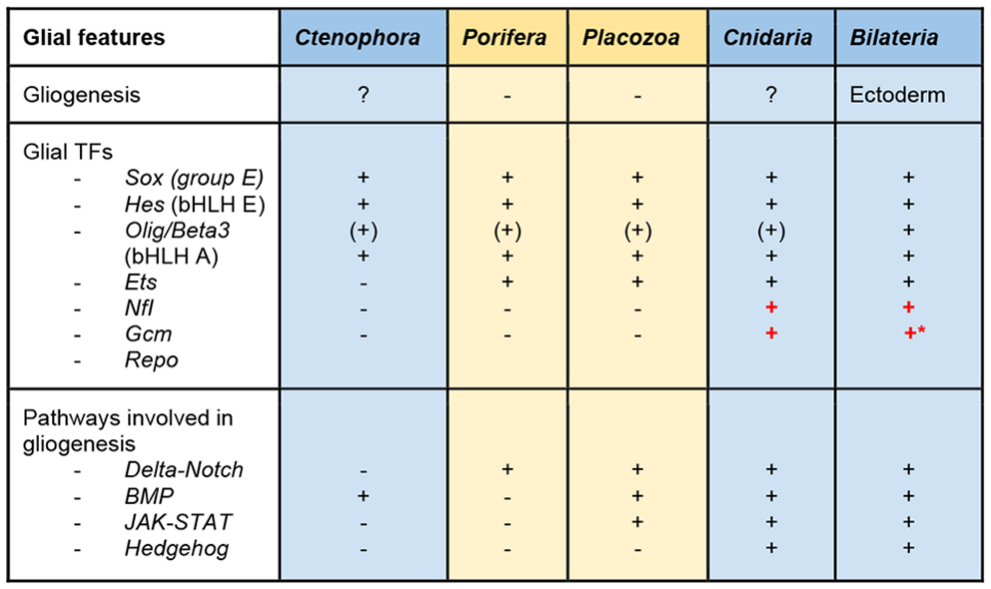
Glial genes in metazoans. Nerveless phyla are highlighted in yellow. Phyla possessing neurons are highlighted in blue. Transcription factors that seem to be related to neural sophistication are shown in red. Asterisk: The *Repo* gene is present in some insects, but not in other bilaterians belonging to *Lophotrochozoa* and *Deuterostomia*

#### SoxE

Certain members of the *SoxE* group drive gliogenesis in bilaterians. *SoxE* genes can be found in genomes of non-bilaterians [182–184] (Table 2). In sponges, *SoxE* is expressed in choanocytes - flagellum-containing cells filtering particles out of the water [185]. Endodermal expression of two *SoxE* genes occurs in both *Ctenophora* [182–184] and *Cnidaria* [186–188]. The function of SoxE genes in these early-branching metazoans still remains to be explored, their broad expression pattern in endoderm does not suggest their involvement in development of nervous systems.

#### Group A and E bHLH genes

Well-known bilaterian gliogenic TFs, *Olig* and *Hes*, belong to Groups A and E of the bHLH family, respectively. Several members of Group A (including *Atonal*) and a single member of group E (*Hes/Hey*) were identified in the *Porifera* [189, 190] (Table 2). Poriferan *Atonal* is expressed in putative sensory cells and had strong proneural activity when it was over-expressed in *Xenopus* [191]. O*ligo/Beta3-like* and *Hes* ortholog were also identified in the *Placozoa* [192]. Three *Hes* genes are present in *Ctenophora* genomes (Table 2). Therefore, it is assumed that bHLH Groups A and E emerged during the very early phase of metazoan evolution. However, a significant expansion of these genes occurred in the *Cnidaria*. Thirty Group A genes and eleven *Hes* copies are present in the genome of a sea anemone, *Nematostella. Nematostella* has two *Olig-like* genes and one of them is expressed in the oral region of endoderm [193, 194]. Expression of *Hes* genes varies [186, 195]. Interestingly, one *Hes-like* gene (*Nvhl3*) is strongly expressed in a subset of cells, which is reminiscent of the *Gcm* expression pattern (see below).

#### Ets family

Both vertebrate (*Ets1, Ets2*) and invertebrate (*Pointed*) genes belonging to the *Pointed Ets* group, which contain an N-terminal *Pointed* domain, have a gliogenic function [152, 196]. In *Nematostella*, expression and a gene regulatory network (GRN) of a *Pointed*-containing *Ets* gene (*NvERG*) has been reported [197, 198]. *NvErg* GRN includes, but is not limited to nervous system components, such as *SoxB*, neuropeptides, and other members of the *Ets* family. Among members of an apical pole GRN of *NvErg* are orthologs of bilaterian glial TFs, *Hes*, *SoxE genes*, and *Gcm.* 12 *Ets* genes were identified in *Nematostella*, but expression and functions of most of them are still unknown.

#### Gcm and Repo

**Fig. 7:**
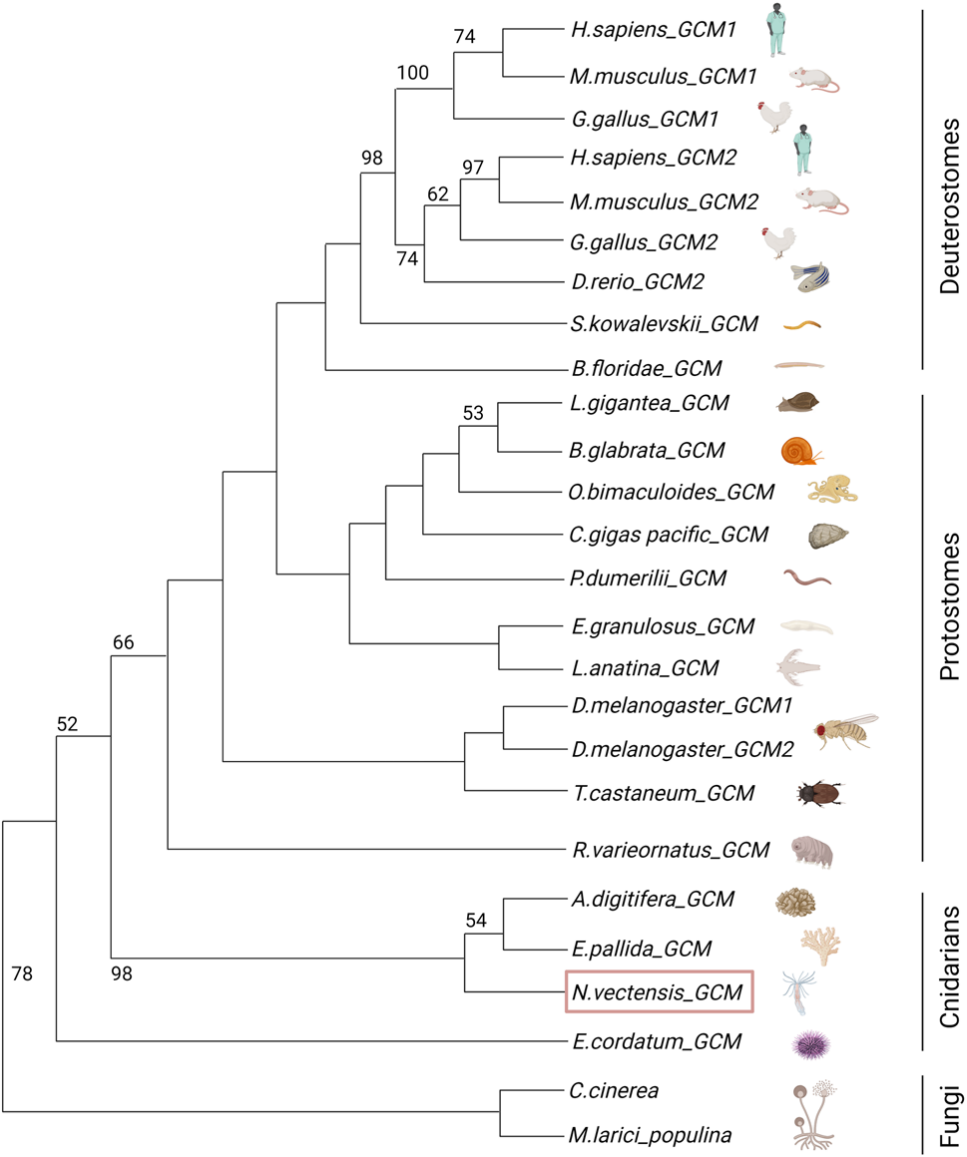
Phylogeny of *Gcm* in metazoans. Among non-bilaterians, *Gcm* is conserved only in the *Cnidaria*. Support values > 50 are shown at basal nodes. *Nematostella Gcm* is framed in red. Species screened for Gcm are listed in Supplementary Table 2. Bidirectional BLAST [199, 200] searches using the *Drosophila melanogaster Gcm1* protein sequence were performed against databases of metazoan organisms. Fungal protein sequences with the highest similarity to animal *Gcm* were used as an outgroup. Sequences were aligned using MUSCLE (Mega7) and trimmed by eye, to include only the domain. The tree was constructed with the maximum likelihood (ML) method using PhyML (SeaView). Bootstrap support is based on 2000 replicates

*Gcm* is a master regulator of gliogenesis in *Drosophila*, which also shows gliogenic potential in vertebrates. Our phylogenetic analysis of *Gcm* revealed that among non-bilaterians, the *Gcm* domain is highly conserved in the *Cnidaria* (Fig. 7, Table S2). *Repo*, another important TF driving gliogenesis under regulation of *Gcm* in *Drosophila*, is also conserved in the *Cnidaria*. In *Nematostella*, *Gcm* is expressed in a subset of cells in the ectoderm at early gastrula stage, and then expands to both endoderm and ectoderm [193, 194]. *Repo* is expressed in the oral region of ectoderm and may participate in specification of the oral nerve ring [193, 194]. Therefore, the two TFs seem to be expressed in different cells, which is unlike *Drosophila Gcm*.

In summary, orthologs of most families containing gliogenic TFs in bilaterians are also present in non-bilaterians (Table 2). However, among non-bilaterians a full complement of bilaterian gliogenic TFs are present only in the *Cnidaria*. Future research focusing on these TF gene regulatory networks and characterizing cells expressing orthologs of bilaterian glial markers in cnidarians should shed light on glial evolutionary roots.

#### Notch-Delta

This pathway is composed of the *Notch* receptor and its ligands (*Delta/Jagged*). Most *Notch-Delta* components are present in *Porifera*, *Placozoa*, and *Cnidaria* (Table 2). This pathway seems functional in all non-bilaterians except the *Ctenophora*, since the latter does not have *Delta/Jagged* [201]. In sponges, this pathway seems to be involved in sensory cell differentiation [191]. In *Nematostella, Notch* signaling regulates neural progenitors and restricts neurogenesis [195, 202, 203]. The function of cnidarian *Notch* is thus reminiscent of bilaterian *Notch*, which acts by repressing neuronal genes. In both *Nematostella* and *Hydra*, *Notch-Delta* is known to regulate development of nematocytes, *Cnidaria*-specific neurosensory cells.

#### Hedgehog

True *Hedgehog (hh)* genes containing both ‘hedge’ and ‘hog’ domains are absent in non-bilaterians, except the *Cnidaria* [204] (Table 2). Hog-domain proteins have been identified throughout the *Metazoa*. In *Nematostella*, *Hedgehog* gene expression analysis shows that true *hh* genes participate in gut formation, and *hh*-related genes are involved in neuronal development [205]. A more recent study demonstrated that germ cell development is dependent on *Hedgehog* in *Nematostella* [206].

#### Jak-Stat

The *Jak-Stat* pathway is composed of several proteins comprising specific domains, which together assemble into a functional system driving transcriptional responses to specific extracellular signals [207]. Non-bilaterian metazoans possess most *Jak-Stat* components (Table 2). The *Ctenophora* has the fewest conserved proteins, while the *Cnidaria* and *Placozoa* only lack one functional unit exclusively present in bilaterians [208]. There are no functional studies of *Jak-Stat* pathway in non-bilaterians.

#### Bmp family

*Bmps* belong to the *Tgf-b* superfamily and are involved in several aspects of neural development, including glial cell differentiation [209, 210]. All early-branching metazoans, except for the *Porifera*, have *Bmp-like* genes [211] (Table 2). In *Cnidaria*, *Bmp* signaling is involved in oral nervous system formation [194]. Given that *Bmp* genes are expressed in a neuron-rich aboral region of ctenophores, they may contribute to nervous system development in these animals as well [211].

**Fig. 8:**
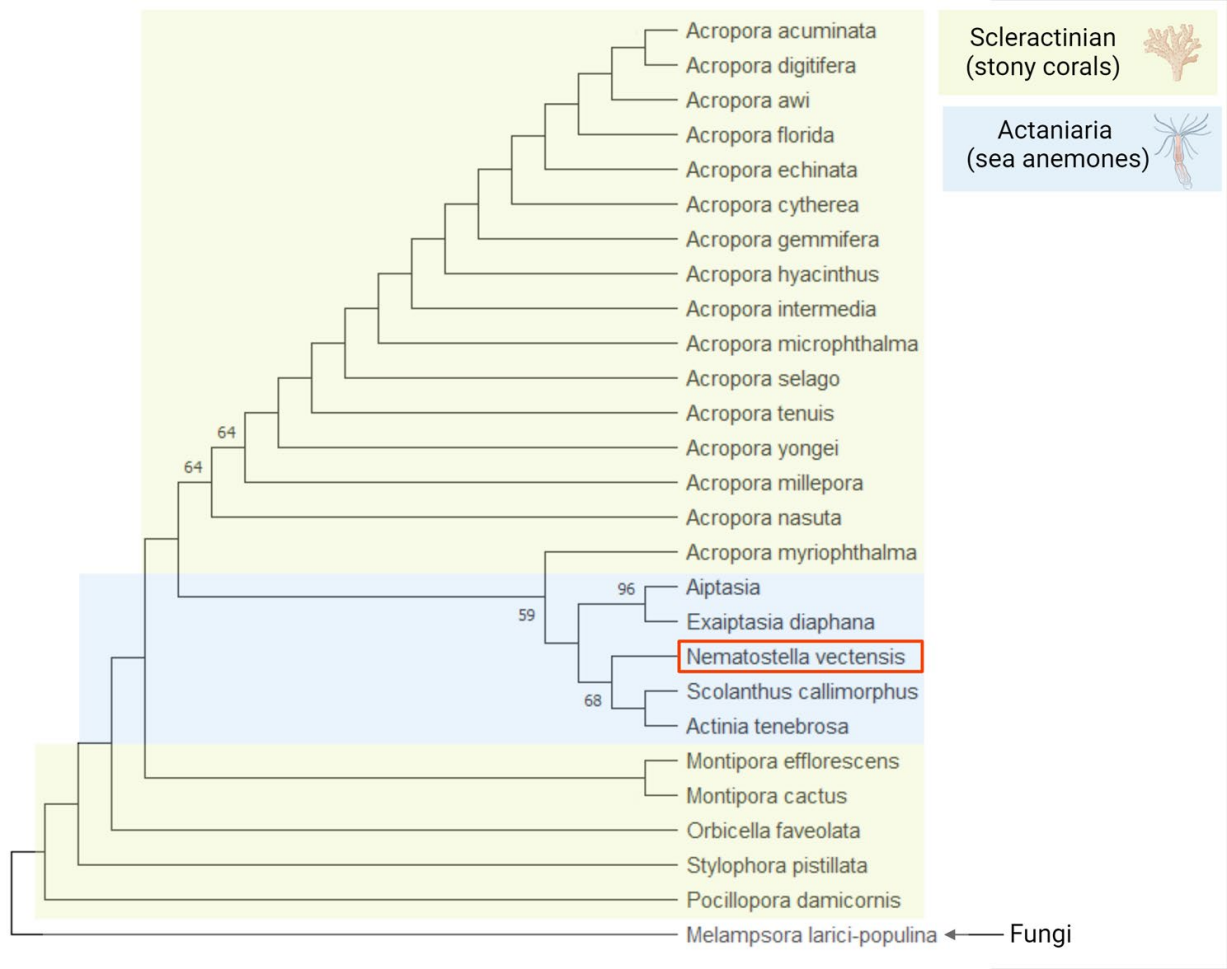
Phylogeny of *Gcm* in *Anthozoa* (*Cnidaria*). This domain is highly conserved in stony corals and sea anemones. Support values > 50 are shown at basal nodes. *Nematostella Gcm* is framed in red. Cnidarian species screened for Gcm are listed in Supplementary Table 3. Bidirectional BLAST [199, 200] searches using *Drosophila melanogaster Gcm1* protein sequence were performed using databases of metazoan organisms. Fungal protein sequences with the highest similarity to animal *Gcm* were used as an outgroup. Sequences were aligned using MUSCLE (Mega7) and trimmed by eye to include only the domain. The tree was constructed with the maximum likelihood (ML) method using PhyML (SeaView). Bootstrap support is based on 2000 replicates

In summary, in addition to gliogenic TF conservation, the *Cnidaria* is the only non-bilaterian phylum characterized by conservation of all signaling pathways required for glial development in bilaterians (Table 2). Moreover, many of these show a conserved function of driving development of the nervous system. Most components of these pathways are present in the other three non-bilaterian phyla, but their functional description is still limited. The *Cnidaria* also possesses all functional glial genes, including GABA and glutamate transporters and enzymes required for their synthesis, glucose transporters, *TRPM* ion channels, aquaporins, etc [178, 212]. Thus, a complete set of neuronal and glial genes in the *Cnidaria* substantiates the possibility of simultaneous evolution of both cell types, as argued by Rey et al., 2020 [9]. Cnidarians are believed to lack glial cells based on morphological assessment of their representatives performed in the 1960s [213]. Horridge and Mackay performed electron microscopic analysis of ectodermal tissue of two cnidarians (jellyfish) and did not observe any cells ensheathing axons or associated with neurons otherwise. In contrast, our phylum-wide genome-wide analysis of bilaterian glial TF conservation in non-bilaterians confirmed that cnidarians have a complete genetic toolkit to drive gliogenesis (Table 2). Therefore, it is imperative to explore this matter in more detail. It is conceivable that if a cnidarian glia population exists, it may consist of just a few cells associated with a particular group of neurons. In addition, the *Cnidaria* consists of two clades, *Anthozoa* (sea anemone, coral) and *Medusozoa* (jellyfish) (Fig. 1), which differ dramatically, not only in their body shape and life cycle, but also genetic composition [214]. Incidentally, *Cnidaria*-specific phylogenetic analysis of *Gcm* revealed that it is highly conserved only in anthozoans (Fig. 8, Table S3). We could not identify *Gcm* orthologs in *Aurelia aurita* (scyphozoan), *Hydra viridissima* (hydrozoan), or *Morbakka virulenta* (cubozoan). Apparently, *Gcm* was lost in the *Medusozoa*, which is consistent with data showing that *Hydra* has lost more transcription factor families than *Nematostella* [215]. The starlet sea anemone, *Nematostella vectensis*, has become an intensely used cnidarian model. This animal is easy to culture in the lab. Its genome, transcriptome, and single-cell transcriptome are available. Numerous gene function manipulation techniques have been developed and transgenic lines of *Nematostella* have been established [178, 212, 216–218]. Accumulating data on neurogenesis, nervous system development and functioning, as well as neuronal type diversity in *Nematostella* make it possible to investigate gliogenic program conservation in this animal. Although no distinct glial cell clusters, or glial transcriptome signatures, have been reported in *Nematostella* [178], further analysis is required to verify the presence of glial function in pre-bilaterian animals. In *Nematostella*, a *Gcm* ortholog is expressed in a subset of cells during development [193]. Our recent studies in *Nematostella* demonstrated that *Gcm* controls expression of *Eaat1*, an astrocyte marker in bilaterians, which is expressed in supposedly neural cells with non-typical morphology [219]. Future experiments including electrophysiology, loss of function, and reporter lines should clarify genetic and physiological features of these cells.

**Fig. 9:**
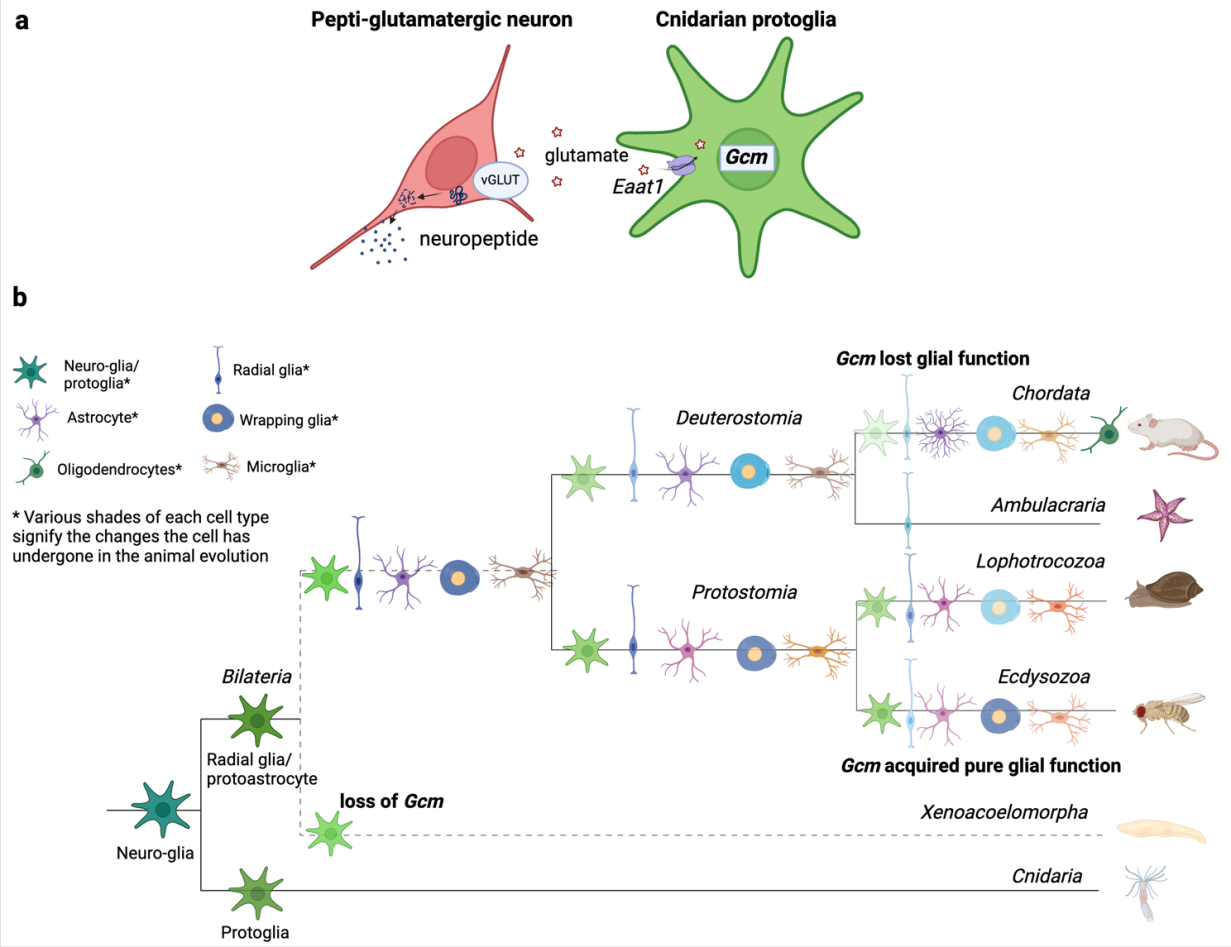
Protoglia function and evolutionary development. (**a**) Schematic representation of cnidarian porotoglia and its interaction with neurons that use both glutamate and neuropeptides as neurotransmitters. (**b**) Evolutionary tree of glia based on the analysis of the gliogenic program conservation described in this review. The diversification of protoglia into various glial cell types with specialized functions is shown

The Nematostella Gcm/Eaat1-expressing cells could represent “protoglia”, which has an important function of glutamate quenching for pepti-glutamatergic neurons (Fig. 9a). These cells could have complementarily combined the features of both glia and neurons and diversified into the distinct cell types later in evolution. The protoglial ancestral cell type had a specific molecular signature that can be traced in Nematostella and is conserved to a various degree in different bilaterian lineages. With functional segregation the Gcm-controlled program either maintained its glial regulation, which is the case in Drosophila, or was significantly modified to have only a potential to induce glial markers as is the case in vertebrates (Fig. 9b). The program kept being modified so that it is almost impossible to recognize it in the most advanced species. This explains the absence of obvious homologous glial cell types in Nematostella. Instead Gcm/Eaat1-expressing cells are a subset of neurons possessing glial features. Metabolic support of neurons, i.e. glutamate recycling, could have been the primary glial function that gave rise to a distinct cell type and separated glia from neurons.

## Conclusions

In recent years, non-bilaterian animals have been extensively studied particularly in the context of neuronal development thanks to rapid development of ‘omics’ tools. However, glial program conservation has not been investigated in these animals. In this review, a comparative analysis shows that all constituents of the bilaterian gliogenic program are conserved in the *Anthozoa* (*Cnidaria*). A representative of this group, *Nematostella vectensis*, could be a useful model system to investigate functions of genes driving gliogenesis in bilaterians and to answer important questions about primordial glia. Until now, no cell clusters with a glial transcriptome signature have been identified in *Nematostella*. This is not surprising, given that no universal glial markers or unified glial genetic signatures are present among bilaterians. Instead, an organism-wide search of glial orthologs and clarification of their functions might reveal novel cell types in the sea anemone. Future studies should clarify functions of conserved bilaterian glial TFs and functional genes not only in *Cnidaria*, but also other non-bilaterians. This is paramount to reconstruct a more accurate picture of glial evolution.

### Electronic supplementary material

Below is the link to the electronic supplementary material.

Supplementary Material 1

## Data Availability

All data generated or analyzed during this study are included in this published article and its supplementary information files.

## Abbreviations

Aldh1l1: Aldehyde dehydrogenase 1 family member L1
Aqp4: Aquaporin channel 4
BBB: Blood-brain barrier
bHLH: Basic helix-loop-helix
BMP: Bone morphogenetic protein
Ced: Cell death abnormality
CNS: Central nervous system
Eaat: Excitatory amino acid transporter
ECM: Extracellular matrix
Ef1a: Elongation factor 1-alpha
Est: Erythroblast transformation specific
GABA: Gamma-Aminobutyric acid
Gapdh: Glyceraldehyde 3-phosphate dehydrogenase
Gat: GABA transporter
Gcm: Glial cells missing
GDNF: Glial-derived neurotrophic factor
Gfap: Glial fibrillary acidic protein
Glut: Glucose transporter
GPCR: G protein-coupled receptor
GS: Glutamine synthetase
Hes: Hairy and enhancer of split
Jak-Stat: Janus kinase-signal transducer and activator of transcription
MMP: Matrix metalloproteinase
NfI: Nuclear factor I
NO: Nitric oxide
NS: Nervous system
Nv: Nematostella vectensis
OPC: Oligodendrocyte precursor cell
Olig: oligodendrocyte transcription factor
PDGFR: platelet-derived growth factor receptor alpha
PNS: peripheral nervous system
Repo: reversed polarity
RGC: radial glial cell
TF: transcription factor
