# Supplementary material for: Evolution of glial cells: a non-bilaterian perspective
Source: Neural Dev. 2024 Jun 21;19:10. doi: 10.1186/s13064-024-00184-4 (PMC11193209; doi:10.1186/s13064-024-00184-4)
Supplement: Supplementary file 1 — Supplementary Material 1 [file 13064_2024_184_MOESM1_ESM.docx]

## **Table S1. Species screened for glial markers.**

| **Phylum** | **Species** | **Glial markers** | **References** |
| --- | --- | --- | --- |
| Vertebrata | *Mus musculus* | *Gfap, vimentin, S100b,*  *Gs, Eaat, Gat, Olig* | Cahoy, J. D. et al. A transcriptome database for astrocytes, neurons, and oligodendrocytes: a new resource for understanding brain development and function. J. Neurosci. 28, 264–278 (2008) |
|  | *Danio rerio* | *Gfap, Gs, Eaat* | Grupp, L., Wolburg, H. & Mack, A. F. Astroglial structures in the zebrafish brain. J. Comp. Neurol. 518, 4277–4287 (2010); Jurisch-Yaksi, N., Yaksi, E. & Kizil, C. Radial glia in the zebrafish brain: Functional, structural, and physiological comparison with the mammalian glia. Glia 68, 2451–2470 (2020) |
|  | *Xenopus laevis* | *Vimentin, Gfap* | Yoshida, M. Glial-defined boundaries in Xenopus CNS. Dev. Neurosci. 23, 299–306 (2001);  D’Amico, L. A., Boujard, D. & Coumailleau, P. Proliferation, migration and differentiation in juvenile and adult Xenopus laevis brains. Brain Res. 1405, 31–48 (2011) |
|  | *Petromyzon marinus* | *Olig1/2, Aldh1l1, Gfap* | Weil, M.-T. et al. Axonal ensheathment in the nervous system of lamprey: Implications for the evolution of myelinating glia. J. Neurosci. (2018) doi:10.1523/JNEUROSCI.1034-18.2018;  Yuan, T., York, J. R. & McCauley, D. W. Gliogenesis in lampreys shares gene regulatory interactions with oligodendrocyte development in jawed vertebrates. Dev. Biol. 441, 176–190 (2018); |
| **Cephalochordata** | *European amphioxus* | *Eaat, Gs, Gfap* | Bozzo, M. et al. Amphioxus neuroglia: Molecular characterization and evidence for early compartmentalization of the developing nerve cord. Glia (2021) doi:10.1002/glia.23982 |
| **Urochordata** | *Ciona intestinalis* | *Gonadotropin-releasing hormone (GnRH)* | Okawa, N. et al. Cellular identity and Ca2+ signaling activity of the non-reproductive GnRH system in the Ciona intestinalis type A (Ciona robusta) larva. Sci. Rep. 10, 18590 (2020) |
| **Echinodermata** | *Asterias rubens* | *If, sspo* | Helm Conrad et al. Early evolution of radial glial cells in Bilateria. Proceedings of the Royal Society B: Biological Sciences 284, 20170743 (2017) |
|  | *Ophioderma brevispinum* | *sspo* | Mashanov, V. & Zueva, O. Radial Glia in Echinoderms. Dev. Neurobiol. 79, 396–405 (2019) |
| Hemichordata | *Balanoglossus misakiensis* | *sspo* | Helm Conrad et al. Early evolution of radial glial cells in Bilateria. Proceedings of the Royal Society B: Biological Sciences 284, 20170743 (2017) |
| **Annelida** | *Magelonidae (mirabilis and alleni)* | *If* | Beckers, P., Helm, C. & Bartolomaeus, T. The anatomy and development of the nervous system in Magelonidae (Annelida) – insights into the evolution of the annelid brain. BMC Evolutionary Biology 19, (2019) |
|  | *Owenia fusiformis* | *sspo* | Helm Conrad et al. Early evolution of radial glial cells in Bilateria. Proceedings of the Royal Society B: Biological Sciences 284, 20170743 (2017) |
|  | *Eisenia fetida* | *Gfap, S100* | Csoknya, M., Dénes, V. & Wilhelm, M. Glial cells in the central nervous system of earthworm, Eisenia fetida. Acta Biol. Hung. 63 Suppl 1, 114–128 (2012) |
|  | *Hirudo medicinalis* | *Gfap* | Riehl, B. & Schlue, W. R. Morphological organization of neuropile glial cells in the central nervous system of the medicinal leech (Hirudo medicinalis). Tissue Cell 30, 177–186 (1998) |
| **Platyhelminthes** | *Schmidtea mediterranea* | *IF, Eaat, Gat, Glut, Gs* | Wang, I. E., Lapan, S. W., Scimone, M. L., Clandinin, T. R. & Reddien, P. W. Hedgehog signaling regulates gene expression in planarian glia. Elife 5, (2016) |
|  | *Christianella minuta* | *S100b* | Biserova, N. M., Gordeev, I. I., Korneva, J. V. & Salnikova, M. M. Structure of the glial cells in the nervous system of parasitic and free-living flatworms. Biology Bulletin 37, 277–287 (2010) |
| **Mollusca** | *Aplysia californica* | *Gs, Ag* | Levenson, J. et al. Long-term regulation of neuronal high-affinity glutamate and glutamine uptake in Aplysia. Proc. Natl. Acad. Sci. U. S. A. 97, 12858–12863 (2000) |
|  | *Octopus vulgaris* | *Gfap, vimentin* | Cardone, B. & Roots, B. I. Comparative immunohistochemical study of glial filament proteins (glial fibrillary acidic protein and vimentin) in goldfish, octopus, and snail. Glia 3, 180–192 (1990) |
|  | *Megalobulimus abbreviatus* | *Gfap, vimentin* | Dos Santos, P. C., Gehlen, G., Faccioni-Heuser, M. C. & Achaval, M. Detection of glial fibrillary acidic protein (GFAP) and vimentin (Vim) by immunoelectron microscopy of the glial cells in the central nervous system of the snail Megalobulimus abbreviatus: GFAP and Vim in glial cells of Megalobulimus. Acta Zool. 86, 135–144 (2005) |
| **Arthropoda** | *Drosophila melanogaster* | *Gcm, repo, Eaat, Gs* | Yildirim, K., Petri, J., Kottmeier, R. & Klämbt, C. Drosophila glia: Few cell types and many conserved functions. Glia 67, 5–26 (2019);  Stacey, S. M. et al. Drosophila glial glutamate transporter Eaat1 is regulated by fringe-mediated notch signaling and is essential for larval locomotion. J. Neurosci. 30, 14446–14457 (2010);  Soustelle, L., Besson, M.-T., Rival, T. & Birman, S. Terminal glial differentiation involves regulated expression of the excitatory amino acid transporters in the Drosophila embryonic CNS. Dev. Biol. 248, 294–306 (2002) |
|  | *Panulirus*  *argus* | *Gs* | Linser, P. J., Trapido-Rosenthal, H. G. & Orona, E. Glutamine synthetase is a glial-specific marker in the olfactory regions of the lobster (Panulirus argus) nervous system. Glia 20, 275–283 (1997) |
|  | *Ucides cordatus* | *Gfap* | Florim da Silva, S. et al. Glial fibrillary acidic protein (GFAP)-like immunoreactivity in the visual system of the crab Ucides cordatus (Crustacea, Decapoda). Biol. Cell 96, 727–734 (2004) |
|  | *Pacifastacus leniusculus* | *Gcm* | Junkunlo, K., Söderhäll, K. & Söderhäll, I. A transcription factor glial cell missing (Gcm) in the freshwater crayfish Pacifastacus leniusculus. Dev. Comp. Immunol. 113, 103782 (2020) |
|  | *Macrobrachium rosenbergii* | *S100b* | Allodi, S., Bressan, C. M., Carvalho, S. L. & Cavalcante, L. A. Regionally specific distribution of the binding of anti‐glutamine synthetase and anti‐S100 antibodies and of Datura stramonium lectin in glial domains of the optic lobe of the giant prawn. Glia 53, 612–620 (2006) |
| **Nematoda** | *Caenorhabditis elegans* | *Lin-26, Hlh-17* | Labouesse, M., Hartwieg, E. & Horvitz, H. R. The Caenorhabditis elegans LIN-26 protein is required to specify and/or maintain all non-neuronal ectodermal cell fates. Development 122, 2579–2588 (1996);  Oikonomou, G. & Shaham, S. The glia of Caenorhabditis elegans. Glia 59, 1253–1263 (2011) |
| **Acoela** | *Symsagittifera*  *roscoffensis* | *Gfap* | Bailly, X. et al. The chimerical and multifaceted marine acoel Symsagittifera roscoffensis: from photosymbiosis to brain regeneration. Front. Microbiol. 5, 498 (2014) |

## **Table S2. Species used for *Gcm* phylogenetic analysis**

| **Phylum** | **Species** | **Gene name, Accession ID** | **Source** |
| --- | --- | --- | --- |
| Chordata | *Homo sapiens* | GCM1, EAX04412.1 | GenBank |
|  |  | GCM2, NP004743 | GenBank |
|  | *Mus musculus* | GCM1, EDL26359.1 | GenBank |
|  |  | GCM2, NP_032130 | GenBank |
|  | *Gallus gallus* | GCM1, AAR24261 | GenBank |
|  |  | GCM2, BAD72825 | GenBank |
|  | *Danio rerio* | GCM2, AAI62304 | GenBank |
| Hemichordata | *Saccoglossus kowalevskii* | GCM, NP_001161553 | GenBank |
| Cephalochordata | *Branchiostoma floridae* | GCM, XP_002z591781 | GenBank |
| Arthropoda | *Drosophila melanogaster* | GCM1, AAC46912 | GenBank |
|  | *Drosophila melanogaster* | GCM2, NP_609302 | GenBank |
|  | *Tribolium castaneum* | GCM, EFA04430 | GenBank |
| Mollusca | *Lottia gigantea* | GCM, XP_009048823 | GenBank |
|  | *Biomphalaria glabrata* | GCM, XP_013078709 | GenBank |
|  | *Octopus bimaculoides* | GCM, XP_014777859 | GenBank |
|  | *Crassostrea gigas* | GCM, EKC24558 | GenBank |
| Annelida | *Platynereis dumerilii* | GCM, CCK33024 | GenBank |
| Platyhelminthes | *Echinococcus granulosus* | GCM, CDS20483 | GenBank |
| Brachiopoda | *Lingula anatina* | GCM, XP_013399310 | GenBank |
| Echinodermata | *Echinocardium cordatum* | GCM, BAS66823 | GenBank |
| Tardigrada | *Ramazzottius varieornatus* | GCM, GAU98972 | GenBank |
| Cnidaria | *Acropora digitifera* | GCM, XP_015774704 | GenBank |
|  | *Exaiptasia pallida* | GCM, KXJ08668 | GenBank |
|  | *Nematostella vectensis* | GCM, EDO25565 | GenBank |
| Fungi | *Coprinopsis cinerea okayama* | Hypothetical protein, EAU87004 | GenBank |
|  | *Melampsora larici-populina* | Hypothetical protein, EGG12299 | GenBank |

## **Table S3. Cnidarian species used for *Gcm* phylogenetic analysis.**

| **Species** | **Accession ID** | **Source** |
| --- | --- | --- |
| *Acropora acuminata* | [aacu_s0062.g65.t1](https://marinegenomics.oist.jp/aacu/genesearch/genemodel?gene_modelid=aacu_s0062.g65.t1&project_id=84) | OIST marine genome browser |
| *Acropora awi* | [aawi_s0172.g23.t1](https://marinegenomics.oist.jp/aawi/genesearch/genemodel?gene_modelid=aawi_s0172.g23.t1&project_id=85) | OIST marine genome browser |
| *Acropora cytherea* | [acyt_s0052.g133.t1](https://marinegenomics.oist.jp/acyt/genesearch/genemodel?gene_modelid=acyt_s0052.g133.t1&project_id=86) | OIST marine genome browser |
| *Acropora digitifera* | [adig_s0165.g12.t1](https://marinegenomics.oist.jp/adig/genesearch/genemodel?gene_modelid=adig_s0165.g12.t1&project_id=87) | OIST marine genome browser |
| *Acropora echinata* | [aech_s0121.g25.t1](https://marinegenomics.oist.jp/aech/genesearch/genemodel?gene_modelid=aech_s0121.g25.t1&project_id=88) | OIST marine genome browser |
| *Acropora florida* | [aflo_s0478.g2.t1](https://marinegenomics.oist.jp/aflo/genesearch/genemodel?gene_modelid=aflo_s0478.g2.t1&project_id=89) | OIST marine genome browser |
| *Acropora gemmifera* | [agem_s0269.g18.t1](https://marinegenomics.oist.jp/agem/genesearch/genemodel?gene_modelid=agem_s0269.g18.t1&project_id=90) | OIST marine genome browser |
| *Acropora hyacinthus* | [ahya_s0011.g87.t1](https://marinegenomics.oist.jp/ahya/genesearch/genemodel?gene_modelid=ahya_s0011.g87.t1&project_id=91) | OIST marine genome browser |
| *Acropora intermedia* | [aint_s0197.g10.t1](https://marinegenomics.oist.jp/aint/genesearch/genemodel?gene_modelid=aint_s0197.g10.t1&project_id=92) | OIST marine genome browser |
| *Acropora millepora* | XP_029180379 | GenBank |
| *Acropora muricata* | [amur_s0056.g15.t1](https://marinegenomics.oist.jp/amur/genesearch/genemodel?gene_modelid=amur_s0056.g15.t1&project_id=94) | OIST marine genome browser |
| *Acropora myriophthalma* | [amic_s0106.g13.t1](https://marinegenomics.oist.jp/amic/genesearch/genemodel?gene_modelid=amic_s0106.g13.t1&project_id=93) | OIST marine genome browser |
| *Acropora nasuta* | [anas_s0200.g8.t1](https://marinegenomics.oist.jp/anas/genesearch/genemodel?gene_modelid=anas_s0200.g8.t1&project_id=95) | OIST marine genome browser |
| *Acropora selago* | [asel_s0087.g22.t1](https://marinegenomics.oist.jp/asel/genesearch/genemodel?gene_modelid=asel_s0087.g22.t1&project_id=96) | OIST marine genome browser |
| *Acropora tenuis* | [aten_s0286.g9.t1](https://marinegenomics.oist.jp/aten/genesearch/genemodel?gene_modelid=aten_s0286.g9.t1&project_id=97) | OIST marine genome browser |
| *Acropora yongei* | [ayon_s0143.g10.t1](https://marinegenomics.oist.jp/ayon/genesearch/genemodel?gene_modelid=ayon_s0143.g10.t1&project_id=98) | OIST marine genome browser |
| *Actinia tenebrosa* | XP_031557567 | GenBank |
| *Aiptasia* | AIPGENE4948 sp\|Q9VLA2\|GCM2 | Reef Genomics database |
| *Nematostella vectensis* | EDO25565 | GenBank |
| *Exaiptasia diaphana* | KXJ08668 | GenBank |
| *Scolanthus callimorphus* | N/A | SIMRbase |
| *Montipora cactus* | [mcac_s0146.g32.t1](https://marinegenomics.oist.jp/mcac/genesearch/genemodel?gene_modelid=mcac_s0146.g32.t1&project_id=100) | OIST marine genome browser |
| *Montipora efflorescens* | [meff_s0379.g17.t1](https://marinegenomics.oist.jp/meff/genesearch/genemodel?gene_modelid=meff_s0379.g17.t1&project_id=101) | OIST marine genome browser |
| *Orbicella faveolata* | XP_020628548 | GenBank |
| *Pocillopora damicornis* | pdam_00014897-RA | Reef Genomics database |
| *Stylophora pistillata* | SpisGene9252 | Reef Genomics database |
